# 固相萃取材料在金属离子前处理应用中的研究进展

**DOI:** 10.3724/SP.J.1123.2020.07004

**Published:** 2021-05-08

**Authors:** Shige XING, Muyi HE, Tong LIU, Wei YONG, Feng ZHANG

**Affiliations:** 中国检验检疫科学研究院食品安全研究所, 北京 100176; Institute of Food Safety, Chinese Academy of Inspection and Quarantine, Beijing 100176, China; 中国检验检疫科学研究院食品安全研究所, 北京 100176; Institute of Food Safety, Chinese Academy of Inspection and Quarantine, Beijing 100176, China; 中国检验检疫科学研究院食品安全研究所, 北京 100176; Institute of Food Safety, Chinese Academy of Inspection and Quarantine, Beijing 100176, China; 中国检验检疫科学研究院食品安全研究所, 北京 100176; Institute of Food Safety, Chinese Academy of Inspection and Quarantine, Beijing 100176, China; 中国检验检疫科学研究院食品安全研究所, 北京 100176; Institute of Food Safety, Chinese Academy of Inspection and Quarantine, Beijing 100176, China

**Keywords:** 固相萃取, 金属离子, 纳米材料, 高分子聚合物, 综述, solid phase extraction (SPE), metal ion, nanomaterial, polymer material, review

## Abstract

为避免摄入过量重金属、危害人类健康,应提高对金属离子的检测能力。常用的金属检测技术如电感耦合等离子体质谱、电热原子吸收光谱、火焰原子吸收光谱等可以有效识别痕量重金属,并且具有多组分分析能力以及检出限低、产量高等优点。但复杂样品本身浓度较低且基质干扰大,因此检测前需进行前处理以消除基质干扰,满足低浓度和小体积样品的检测需求。固相萃取是富集样品中金属离子常用的方法之一,开发能够进行高效、快速富集分离的固相萃取新材料及前处理技术是金属离子检测的关键。限制接触碳纳米管、纳米吸附剂、纳米粒子载体、磁性纳米粒子等纳米材料可提供大的比表面积和可调的官能团,以促进金属离子吸收,其优越的光学性能则可用于荧光和比色检测;高分子聚合物具有卓越的机械性能和化学稳定性,可用于微量金属粒子的前富集、分离和检测;离子印迹聚合物对目标离子具有选择性识别能力的空间结构,可以吸附待分离体系中的金属离子;双功能材料可同时进行多种金属离子的萃取和快速定量检测,新型的光敏络合物则可以将结合态的金属离子转变为游离态,使其被多种生物传感器快速检测,也可以研究生物体内金属离子的信号传递过程。该文综述了纳米材料、聚合物、功能材料等新型固相萃取材料的特点及在复杂样品前处理中的应用和研究进展,并对其未来发展方向进行了展望。

## Abstract (en)

Monitoring of trace heavy metal pollutants released during industrial and agricultural processes is essential because of their widespread distribution in the environment and health hazards. Several techniques, including inductively coupled plasma-mass spectrometry (ICP-MS), inductively coupled plasma-optical emission spectrometry (ICP-OES), electrothermal atomic absorption (ETAAS), and flame atomic absorption spectrometry (FAAS), have been proposed for the determination of heavy metals in serum, plasma, whole blood, and food. All these techniques have earned robust recognition in the field of trace heavy metals and have many advantages such as multi-elemental analysis capability, large dynamic linear range, low detection limits, and high productivity. Nevertheless, most of the recommended techniques require digestion of the sample and extraction with an organic solvent for isolation of the metal ion from the sample solution prior to analysis. Despite improvements in the performance of modern analytical instruments, the direct determination of heavy metal ions in real samples is difficult because of their low concentration levels and matrix interference. Thus, extraction and clean-up steps are required for pre-concentration of the analyte, so that detection and elimination of the interfering matrix component are possible. Solid-phase extraction (SPE) is one of the popular metal ion pretreatment methods. The advantages of SPE include easy cartridge/column regeneration, high analytical frequency, and high preconcentration factors for sorbents with high adsorption capacities. On the other hand, when the analytes are extracted from a complex matrix such as serum and meat samples, large amounts of proteins from the samples can be retained on the sorbent surface, obstructing the binding sites on the sorbent and leading to poor precision and accuracy. The key to metal ion detection is the development of new SPE materials with high efficiency and enrichment factors as well as an effective pretreatment technology. Nanomaterials such as restricted-access carbon nanotubes, nanoadsorbents, nanoparticle carriers, and magnetic nanoparticles have shown great promise in advancing biomedical and environmental analysis because of the unique properties originating from their ultrafine dimensions. Nanomaterials can provide large specific surface areas and tunable functional groups to facilitate metal ion absorption. They could also possess superior optical properties and allow for high sensitivity in simple fluorescent or colorimetric detection methods. Owing to their excellent mechanical and chemical stability, polymer materials have been of great interest as adsorbents for the SPE of metal ions from solution. Moreover, a designed polymeric material can show triple functionality such as physical adsorption, chelate formation, and ion exchange for the target metal ions. A dual-functional nanomaterial-DNAzyme platform can simultaneously allow for the sensitive detection and effective removal of heavy metal ions in water. Thus, this platform can serve as a simple, cost-effective tool for rapid and accurate metal quantification in the determination of human metal exposure and inspection of environmental contamination. Furthermore, the new photocaged chelator can uncage and release the combined metal ions into an aqueous solution that is free of the other components of the matrix. In this manner, we can develop diagnostic tests for metal ions that are often difficult to detect using other methods. In this paper, the characteristics of new SPE materials, including nanomaterials, polymer materials, and functional materials as well as advances in their applications to the preparation of complex samples are summarized, and the direction for future development is proposed.

金属离子广泛存在于环境中,环境和食品中的金属污染会对人类构成巨大威胁,导致人们产生严重的健康问题,如失忆、失明和失聪、肾脏损伤和患癌等^[1,2]^。因此人们应避免摄入过量的重金属,同时需加强对重金属的风险检测和风险管理。

目前常用的金属检测技术包括电感耦合等离子体质谱(ICP-MS)、电感耦合等离子体光发射光谱(ICP-OES)、电热原子吸收光谱(ETAAS)、火焰原子吸收光谱(FAAS)等^[3,4,5,6]^。虽然这些检测技术可有效识别痕量重金属,并且具有多组分分析能力,以及动态线性范围大、检出极限低、产量高等优点。但是复杂样品如食品和生物溶液中重金属的检测仍存在很多瓶颈,主要原因在于样品本身的浓度水平较低以及基质干扰较大。因此需要对重金属进行提取、净化等预富集前处理过程,以消除基质成分的干扰,满足低浓度和小体积样品的检测需求。

目前常见的样品前处理方法有液相萃取(LPE)、固相萃取(SPE)、液固萃取(L-SPE)、超临界萃取(SFE)等。SPE的萃取剂为固体,作为样品前处理的一个常用方法,其在金属离子的预富集中应用广泛^[7,8]^。SPE具有易于再生和分析频率高的优点,对于吸附能力强的吸附剂(如碳纳米管(CNTs))有较高的预富集因数。但是,传统的SPE方法需要多个萃取和清洗步骤,因此存在烦琐、耗时、稀释比例高等缺点。另外,当从蛋白溶剂(如血液、血浆、血清)中提取金属离子时,样品中大量的蛋白质会保留在吸附剂表面,阻碍吸附剂的结合位点,导致结果的不精确和不准确。近年来,在传统SPE的基础上发展了微固相萃取(μ-SPE)、分散-微固相萃取(D-μSPE)、固相微萃取(SPME)等方法。开发能够高效、快速富集分离的固相萃取新材料及前处理技术是一个非常重要的研究课题。

## 1 纳米材料

纳米材料由于其独特的尺寸和理化性质在样品前处理、信号标记等方面具有较多优势。纳米材料的表面性质使其能够通过各种戴帽配体实现功能化,经过精心设计,可以提供大的比表面积和可调官能团,促进金属离子吸附。它们还具有优越的光学性能(如淬灭能力),可用于进行简单且灵敏度高的荧光和比色检测^[9]^。因此,纳米粒子在物理、化学、生物、医学等领域得到了广泛关注^[10,11]^,也广泛应用于金属离子固相萃取中。

### 1.1 限制接触碳纳米管

Barbosa等^[12,13]^将氧化后的CNTs表面覆盖上牛血清白蛋白(BSA)层,形成限制接触碳纳米管(RACNTs)。以戊二醛为交联剂,BSA的氨基相互交联固定在CNT表面,当生物液体在高于蛋白质等电点的pH值下通过RACNTs柱时,样品和BSA层的蛋白质都带负电荷,引起静电排斥,因此该材料可以将Cd(Ⅱ)、Pb(Ⅱ)从血清中萃取出来(见图1),最大吸附量为34.5 mg/g。研究者^[12]^将填充有RACNTs的微柱用于在线SPE系统,并且偶联FAAS进行金属离子检测。在优化后的实验条件下,该方法的检出限可达2.1 μg/L,富集因子为5.5,日内和日间的精密度<8.1%,未经处理人血清中Pb(Ⅱ)的加标回收率为89.4%~107.3%。该RACNTs微柱可进行超过200次萃取,而不丢失萃取效率,并且Pb(Ⅱ)含量保持在μg/L水平。RACNTs具有对样品量的限制少、灵敏度和准确度好、分析频率高,以及试剂和样品消耗量少的优点。RACNTs还可用于复杂样品中直接萃取无机和有机分子。

**图 1:**
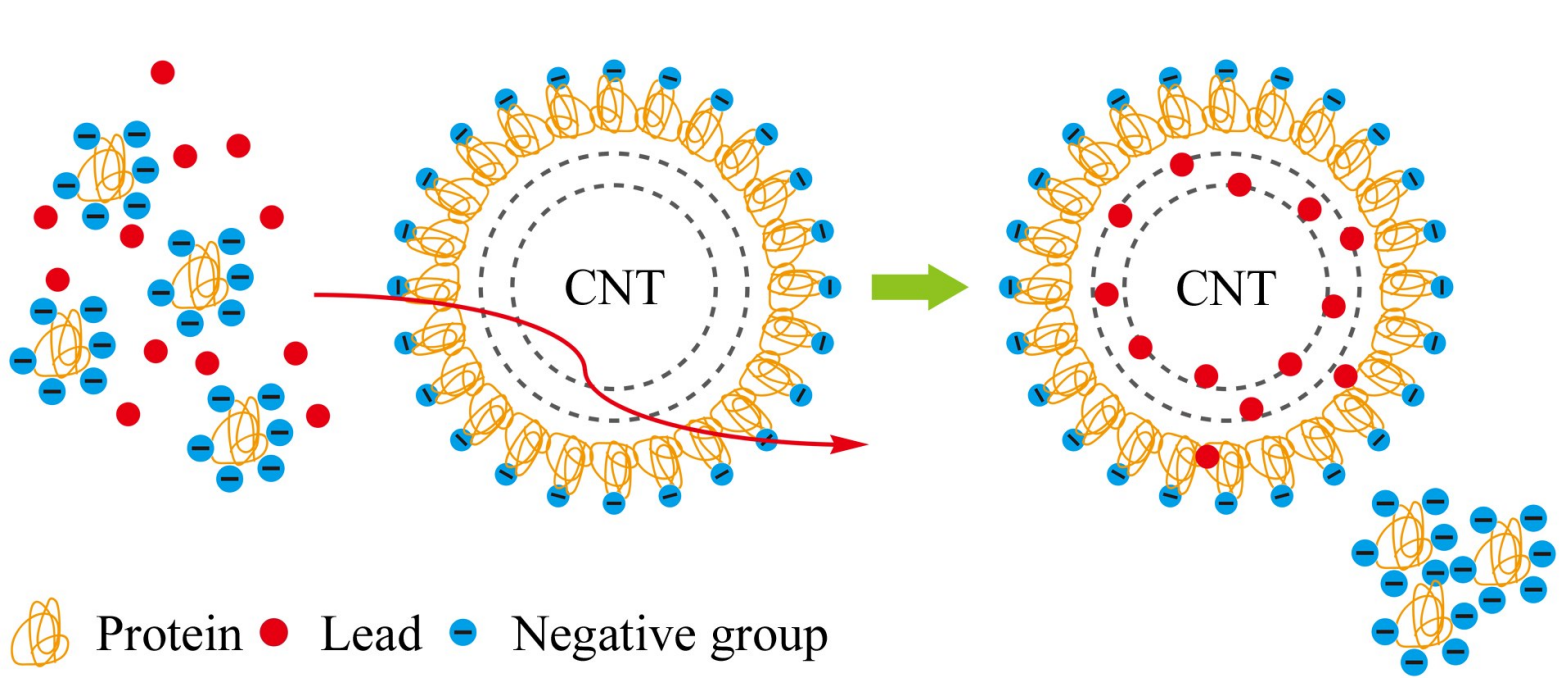
RACNTs微柱萃取Pb(Ⅱ)示意图

### 1.2 纳米吸附剂

最近,μ-SPE作为SPE的微型形式被发展用于低溶剂、吸附剂消耗的样品处理中。吸附剂驻留在一个密封的多孔膜包膜中,并保护其不受样品基体的影响。该方法能同时进行样品清理和提取,适用于复杂基质样品^[14]^。μ-SPE方法基质效应不明显,商品化吸附剂类型多,因此应用广泛。在一种改进的D-μSPE方法中,萃取发生在整体溶液中,因而吸附剂能与所有目标物快速均匀的相互作用^[3,15]^。

Ghazaghi等^[16]^将D-μSPE纳米吸附剂用于富集Pb(Ⅱ)和Cd(Ⅱ)。他们通过化学气相沉积将石墨烯沉积在斜发沸石上制备石墨烯-斜发沸石(G-CL)混合纳米吸附剂。结构表征显示,石墨烯片沉积在沸石的多孔结构周围,具有高表面积。这些石墨烯片充当了屏障以阻挡样品基质中可能存在的大分子,因而金属离子可穿透斜发沸石的多孔结构,而部分与蛋白质结合的金属离子则吸附在石墨烯表面,将游离和结合的金属离子提取出来。该纳米材料可作为吸附剂用于分离和预浓缩生物样品中痕量Pb(Ⅱ)和Cd(Ⅱ)。这种小的提取单元可将清洗和富集步骤同时进行,方便处理小样本容量,因此也增加了预浓缩系数。

G-CL纳米材料对金属离子的吸附原理可能是基于静电力,其受到吸附剂用量、洗脱浓度、洗脱体积及超声波浴时间等因素的影响,并且与溶液pH值密切相关。在较低pH值时,质子和分析物竞争占据活性位点,回收率降低;较高pH值时,两种金属离子和-OH形成沉淀,导致回收率降低。实验数据显示,当溶液pH=5、反应时间30 s时,金属离子的萃取回收率为97%。将该方法与电热原子吸收光谱(ET-AAS)联用,测定水和人血清中Pb(Ⅱ)和Cd(Ⅱ)的检出限分别为70 ng/L和4 ng/L。实际样品检测结果显示,在各类水样及类似血清的复杂基质中,该方法的回收率为92.3%~99.0%。

### 1.3 纳米粒子载体

Abdolmohammad-Zadeh等^[17]^以8-羟基喹啉(8-HQ)功能化CoFe_2_O_4_纳米粒子为载体,以十二烷基硫酸钠(SDS)为辅助物,建立了简单固相微萃取分离水溶液中Al(Ⅲ)离子的方法。8-HQ是一种非常灵敏的荧光检测配体,可以和Al(Ⅲ)离子结合形成高荧光信号的复合物^[18]^。与8-HQ/SDS/CoFe_2_O_4_纳米粒子相互作用的荧光强度相比,Al(Ⅲ)-8-HQ络合物在本体溶液中的荧光强度提高了近5倍(见图2)。在优化条件下,校准曲线的线性范围为0.1~300 ng/mL,相关系数为0.9986,检出限和定量限分别为0.03 ng/mL和0.10 ng/mL。该方法可直接测定人体血清和水样中Al(Ⅲ)离子,方法快速、简单,灵敏度高,不需要额外的清洗过程。

**图 2:**
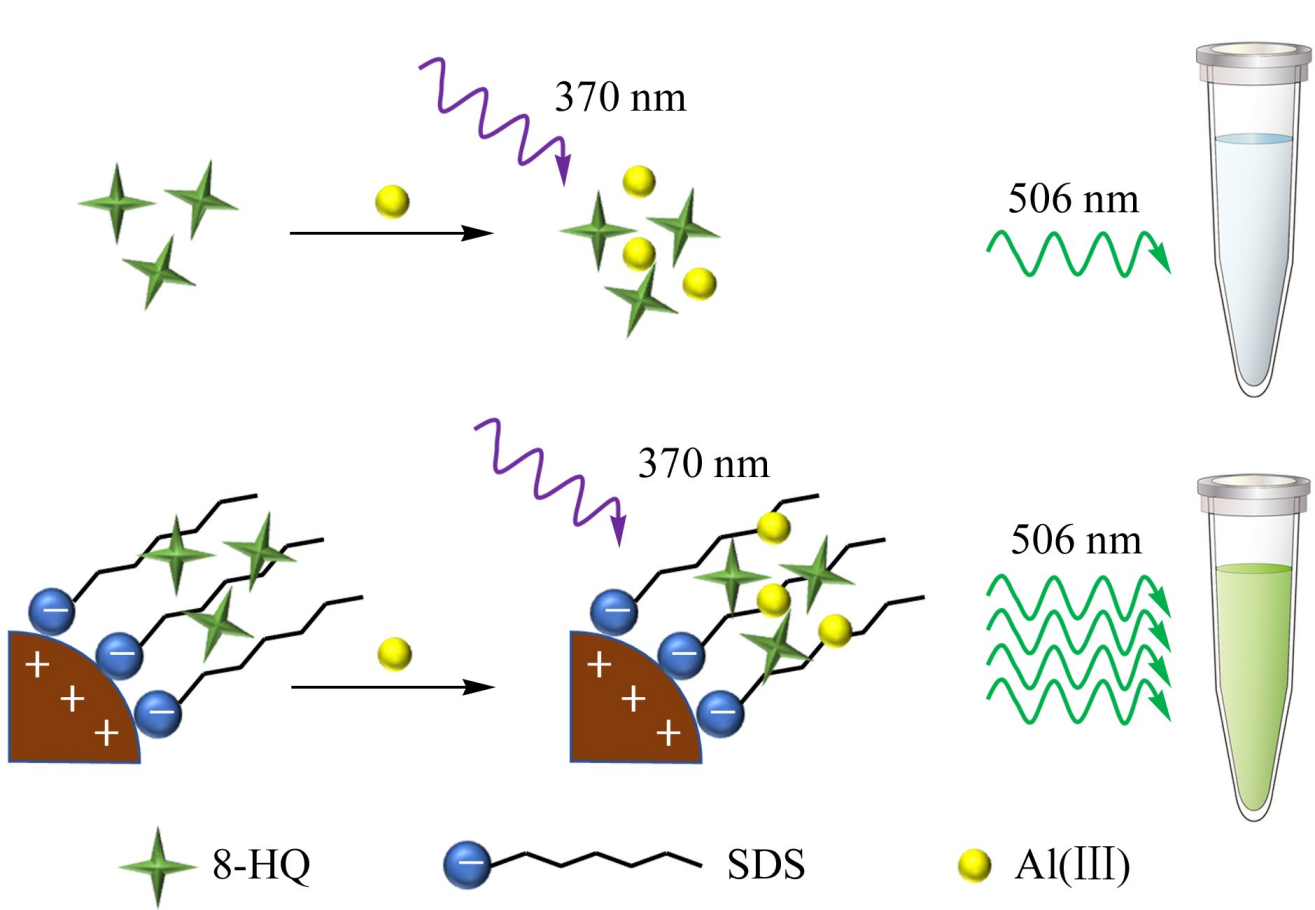
8-HQ功能化CoFe_2_O_4_纳米粒子和Al(Ⅲ)离子结合产生具有较强荧光的Al(Ⅲ)-HQ/SDS/CoFe_2_O_4_纳米粒子复合物示意图

### 1.4 磁性纳米粒子

常规的SPE技术在处理样品时需要进行离心,在离心作用下,有些离子发生共沉淀反应,改变了样品性状,会导致一些干扰和损失。磁性固相萃取(MCPE)是通过磁性材料吸附目标物质,外加磁场使之快速分离的方法^[19,20]^,其示意图见图3^[20]^。利用Fe_3_O_4_制备的功能化磁性纳米材料因具有较强磁性且易通过外部磁场进行固液分离,免去了复杂的过滤和离心过程,在重金属吸附萃取方面起重要作用,其中应用较多的是碳基磁性纳米材料^[21,22]^。

**图 3:**
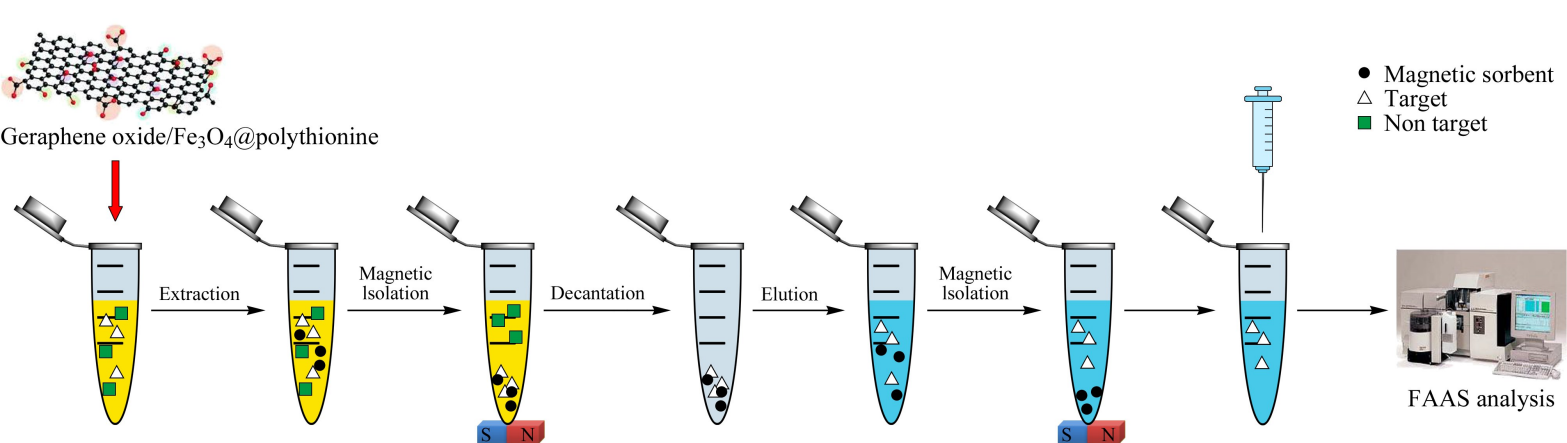
磁性固相萃取示意图^[20]^

Tang等^[23]^将磁性碳纳米管(MCNTs)用于萃取废水中的Cu(Ⅱ),并联用原子吸收光谱进行检测;磁性石墨碳化氮(MC_3_N_4_)也在Zn(Ⅱ)、Cr(Ⅵ)、Cd(Ⅱ)、Pb(Ⅱ)的萃取中有所应用^[24]^;磁性氧化石墨烯(MGO)被用于萃取水中的Zn(Ⅱ)、Cr(Ⅵ)、Cd(Ⅱ),将其与分散液液微萃取结合用于水样中金属离子的富集测定,解决了后者选择性较差的不足^[20,25]^。Filik和Avan^[26]^采用室温离子液体分散液液微萃取与分散磁固相微萃取相结合的两步微萃取技术,添加磁性Fe_3_O_4_纳米粒子作为吸附剂,在有机溶剂中收集分析物,磁固相分离后再用0.1 mol/L HCl洗脱富集物配合物,用于水样中痕量钴离子的FAAS测定。

## 2 聚合材料

### 2.1 高分子聚合物

高分子聚合材料具有卓越的机械性能、热能、化学稳定性和可重复利用性,可作为吸附剂从水溶剂中固相萃取金属离子^[27,28]^。这些聚合物可以用金属鏊合进行功能化,功能化的配体分子可以和聚合材料的活性位点如羟基、羧基、氨基、环氧丙基、吡啶等共价结合,以对金属离子产生更高的选择性和更强的结合性。功能化的多聚物材料以树脂、微球、凝胶的形式作为固相萃取的吸附剂用于微量金属离子的前富集、分离和检测。其中,交联多孔微球是主要的使用形式,其具有比表面积高、多孔性、耐用性、流动性和可回收的特点。环丙异丁烯酸甲酯是一种商业化的工业单体,侧链上有一个环氧基,非常适合制备适宜尺寸的功能性多孔微球,其表面环氧丙基易通过开环反应被各种类型配体修饰^[29]^。因此,选择适宜配体对于获得较高的选择性和容量非常重要。噻唑环中N和S的供体中心表现出对二价过渡金属离子和惰性金属优越的吸附选择性,可以形成复合物,因此噻唑的衍生物是一个可用于多聚体载体功能化的有效多配位配体^[30]^。

多孔交联的多聚缩水甘油酯-甲基丙烯酸甲酯-二乙烯基苯(GMA-MMA-DVB)三元聚合物通过悬浮聚合作用合成微球,经GMA上环氧基的开环反应与2-氨基苯并噻唑(ABTAL)进行功能化修饰合成功能化多聚体微球,多聚GMA-MMA-DVB作为固相吸附剂用于选择性的前富集/分离,联合FAAS检测不同饮料中Al(Ⅲ)、Fe(Ⅱ)、Co(Ⅱ)、Cu(Ⅱ)、Cd(Ⅱ)、Pb(Ⅱ)的含量^[27]^。在优化的条件下,Pb(Ⅱ)的回收率为97%, Al(Ⅲ)、Co(Ⅱ)回收率为98%, Fe(Ⅱ)、Cu(Ⅱ)、Cd(Ⅱ)回收率为99%。该研究证实了在FAAS检测前使用ABTAL功能化微球对样品进行固相萃取的可行性。该分析流程具有较好的富集因数和较低的检出限,且环境污染较少,因而可以简单快速的用于不同类型的饮料前处理中,从而进一步应用于真实样品金属离子含量检测。

### 2.2 离子印迹聚合物(IIPs)

IIPs是一种基于印迹技术的新型吸附剂,具有聚合物基质中的特异性识别位点。离子印迹的原理是在模板离子和适当的配体之间形成复合物,然后在交联剂的存在下进行聚合,经洗脱剂脱除模板离子后形成对目标离子具有选择性识别能力的空间结构,可以吸附待分离体系中的目标物质^[31]^。IIPs对目标离子的特异性吸附亲和力高,不污染样品,因此在金属离子的前处理中应用广泛^[32,33,34]^。

梁维新等^[35]^以Pb(Ⅱ)为模板离子,制备了铅离子印迹聚合物微球(IIPMs)(见图4),填装成固相萃取柱对水样中的Pb(Ⅱ)进行富集,最大富集倍数可达250倍,能重复利用12次以上;同时建立了地表水中痕量Pb(Ⅱ)的印迹聚合物固相萃取-微波等离子体发射光谱(MP-AES)测定方法,在最优萃取条件下,该法的检出限为0.26 μg/L,实际地表水样的加标回收率为92.4%~98.8%,相对标准偏差不大于4.1%。因此可用于地表水中痕量Pb(Ⅱ)的准确测定。

**图 4:**
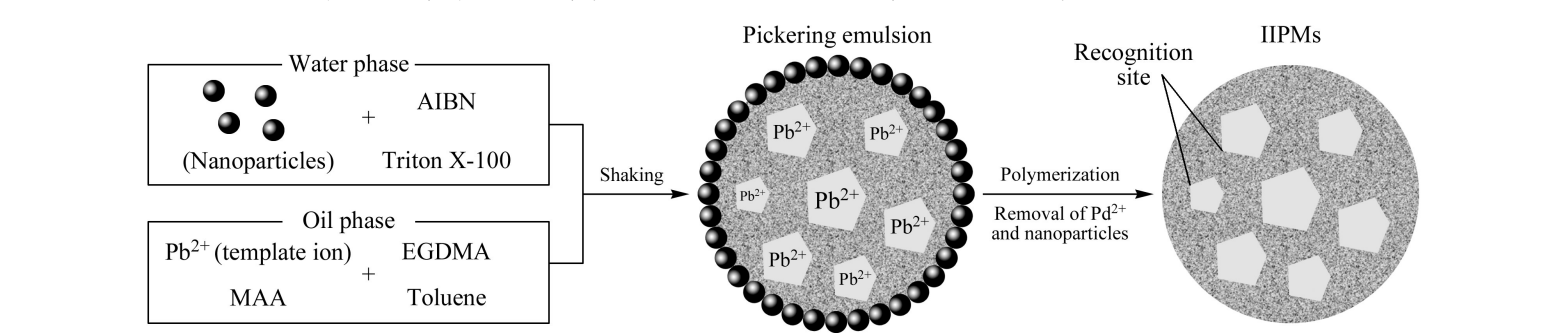
皮克林乳液聚合法合成IIPMs^[35]^

Moussa等^[36]^成功合成了能够同时选择性提取所有镧系的IIPs。在优化的SPE条件下,IIPs对镧系家族8个代表性离子的萃取回收率均高于77%,平均回收率为83%;而非印迹聚合物(NIPs)的萃取回收率则为14%~36%。

## 3 新型功能材料

### 3.1 双功能材料

在复杂的生物或环境样品中,检测金属混合物比单金属检测需要更高的灵敏度和选择性。质谱法和光学光谱法是评估环境中金属混合物的主要方法,但它们昂贵,占用空间较大,使得现场实时探测变得困难。因此迫切需要开发新方法用于重金属污染的现场调查和现场应用。

Fang等^[37]^基于高选择性和高吸收率的金属响应性纳米材料,设计了一种双功能的纳米材料平台,可以同时富集和灵敏检测多种金属离子。该平台由大孔石墨烯泡沫(GF)和金属响应型DNA酶组成。金属响应型DNA酶是通过指数富集配体(SELEX)系统进化筛选的一类特殊酶,其对特定金属离子表现出良好的催化能力和结合活性^[38,39,40]^。如图5所示,GF作为金属离子提取剂和荧光标记DNA酶的萃取剂,GF表面的磷酸基通过金属-磷酸盐配位提取二价金属离子,石墨烯主链可以与单链DNA (ssDNA)紧密结合,并抑制附着在ssDNA上的荧光团^[37]^。一旦Pb(Ⅱ)和Cu(Ⅱ)在GF表面富集,将会激活对应的金属响应DNA酶,释放出两种荧光标记的单链DNA,该荧光标记DNA单链可被GF吸附和淬灭。研究人员通过检测两种荧光强度的减少,实现了两种金属离子的同时定量检测。该方法的回收率为95.0%~98.3%,成功定量了人血清和河水中的Pb(Ⅱ)和Cu(Ⅱ),检出限分别为50 pmol/L和0.6 nmol/L。

**图 5:**
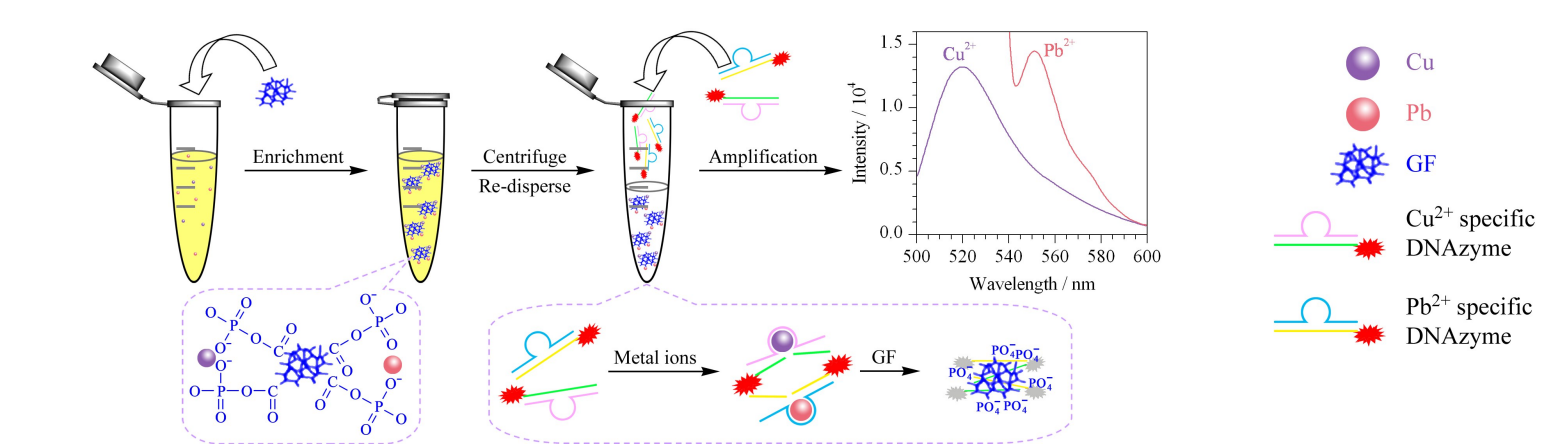
GF和DNA酶为基础的检测金属离子方法示意图^[37]^

磷酸盐修饰的GF可以富集不同类型的二价金属阳离子,利用不同金属特异性DNA酶,结合多种波段的荧光分子,可实现多种金属离子的同时快速定量检测.该双功能平台对重金属污染的现场调查和患者金属暴露的诊断具有重要价值。

### 3.2 光敏金属络合物

近年来,生物传感器(如DNA酶)在环境监测、医疗诊断和成像等领域得到了广泛应用^[41,42]^。但探测金属离子仍然存在一个主要障碍,即传感器无法捕捉与生物分子络合的“结合态”金属。已经开发的检测方法适用于生物分子亲和力较弱的金属离子(如Ca^2+^和Na^+^)^[43]^,但与生物分子紧密结合金属离子的检测灵敏度往往较低^[44]^。因此,如何将结合态的金属离子转变为游离态的形式,一直是金属离子快速检测的一个瓶颈问题。

光敏金属络合物是一种螯合剂,通过光化学反应可调节其结合亲和力。该络合物可捕获溶液中的金属离子,在紫外光照下,络合物与金属离子之间的共价键断裂,螯合效应减少,金属离子随之释放。如图6所示,Basa等^[45,46]^合成了多种金属离子特异性的光敏感螯合物,其中Zn离子特异的光敏感螯合物{双[(2-吡啶基)甲基]氨基}(9-氧-2-黄烯基)乙酸(XDPAdeCage)在紫外光下利用2-黄酮乙酸介导光脱羧反应释放金属离子^[46]^。数据显示XDPAdeCage光解的量子产率为27%,与Zn(Ⅱ)结合的亲和力为4.6 pmol/L,在光解后亲和力降低了4个数量级以上。

**图 6:**
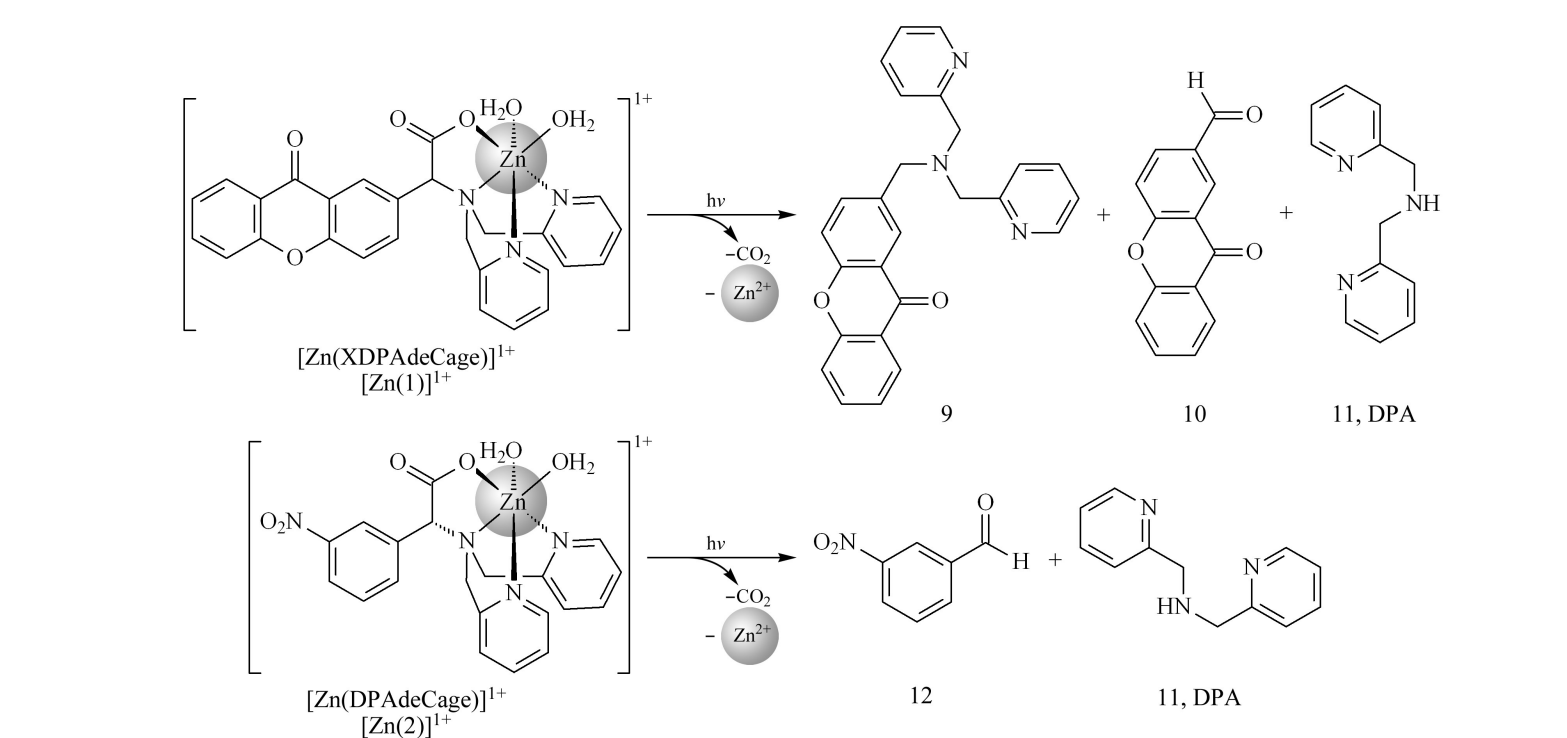
Zn(Ⅱ)离子特异的光敏感螯合物(XDPAdeCage)萃取及释放Zn(Ⅱ)过程示意图^[46]^

使用该种方法可以成功地将生物样品中结合态的Zn(Ⅱ)释放出来并转变为游离态的Zn(Ⅱ),进而可以被多种生物传感器快速检测,也可以研究生物体内(如细胞中)金属离子的信号传递过程。

## 4 总结与展望

重金属污染已引起全社会的广泛关注,其对人体健康和生态环境的危害也日益显现。固相萃取具有回收率和富集倍数较高、易于再生等优点,已成为最常用金属离子前处理方法之一。如何对复杂样品基质中的微量或者痕量金属离子进行富集分离是金属离子前处理中的核心和关键,为了解决这一问题,高效、高选择性的新型固相萃取分离材料的研究发展迅速。

本文综述了限制接触碳纳米管、纳米吸附剂、纳米粒子载体、磁性纳米粒子等纳米材料、高分子聚合物和功能材料的特点及其在生物、环境污染物、食品样品前处理中的应用。这些新型材料具有独特的理化性质,环境友好,对金属离子的特异性高,因而可以快速高效地富集萃取复杂样品中的金属离子,有些还可以同时进行金属离子的萃取和检测,实现了金属离子的现场快速检测。因而在各类复杂样品的前处理过程中有着良好的应用潜力。

总之,日益发展的新型萃取材料将在复杂样品前处理过程中得到越来越多的应用。开发新型萃取材料,发展简便、高效、快速、绿色的金属离子检测方法,以及实现样品前处理与分析同步化、自动化、环境友好化将是未来金属离子检测的发展方向。
